# Inflammatory Hyperreflective Retinal Foci: An OCT Biomarker of Neuroinflammation in Geographic Atrophy

**DOI:** 10.3390/jcm15041453

**Published:** 2026-02-12

**Authors:** Federico Parolini, Elisabetta Pilotto, Edoardo Midena, Giulia Midena

**Affiliations:** 1 Department of Neuroscience-Ophthalmology, University of Padova, 35128 Padova, Italy; federico.parolini@studenti.unipd.it (F.P.); edoardo.midena@unipd.it (E.M.); 2 IRCCS-Fondazione Bietti, 00198 Rome, Italy; giulia.midena@fondazionebietti.it

**Keywords:** age-related macular degeneration, OCT, retinal neuro-inflammation, hyperreflective retinal foci, geographic atrophy, microglia

## Abstract

**Background**: Inflammatory hyperreflective retinal foci (I-HRF) have been recognized as an optical coherence tomography (OCT) biomarker of aggregates of activated microglial cells. Microglia, the principal resident immune cells, are key players in geographic atrophy (GA) development and progression. **Objective**: To quantify I-HRF distribution across inner (IR) and outer (OR) retinal layers in GA compared with healthy controls. **Methods**: Retrospective observational study including patients aged ≥50 years with GA lesion area >1.25 mm^2^ and age-matched healthy subjects. GA eyes were classified as bilateral GA (B-GA) or unilateral GA (U-GA; fellow eye with macular neovascularization). Using Spectralis OCT, I-HRF (≤30 μm; RNFL-like reflectivity; no posterior shadowing) were identified and counted across IR and OR. **Results**: Sixty-eight eyes from 46 patients with GA (B-GA: 49 eyes; U-GA: 19 eyes) and 19 control eyes were studied. I-HRF were higher in IR than in OR in all groups (*p* < 0.001). I-HRF were higher in GA eyes in both layers compared with controls (*p* < 0.05). U-GA exhibited higher I-HRF than B-GA in IR (44.32 ± 8.47 vs. 30.10 ± 7.62; *p* < 0.001), while I-HRF were not significantly different in OR (9.58 ± 3.04 vs. 8.02 ± 3.33; *p* = 0.081). **Conclusions**: I-HRF are increased in GA. They are more numerous in IR, consistent with their proposed inflammatory origin. These findings further support the role microglia may play in GA pathology. I-HRF may become an OCT biomarker to track GA-associated neuroinflammation in different GA phenotypes. Longitudinal studies are needed to clarify I-HRF significance in GA progression.

## 1. Introduction

Geographic atrophy (GA), the advanced atrophic stage of age-related macular degeneration (AMD), is a leading cause of irreversible vision loss in the elderly [1]. Its prevalence is expected to increase sharply as the global population ages [2,3]. GA is characterized by well-defined areas of macular atrophy involving photoreceptors, retinal pigment epithelium and the underlying choriocapillaris layer [4,5]. Functionally, GA areas correlate with dense scotoma by means microperimetry, the spatial extent of which corresponds to the chorioretinal atrophic area [6,7]. GA areas initially occur in the parafoveal retina, sparing in the foveal area. Over time, GA areas enlarge, coalesce, and new atrophic areas may occur. As long as the fovea remains unaffected, high-contrast central Best Corrected Visual Acuity (BCVA) is preserved. However, patients with GA complain of severe impairment in reading performance, contrast sensitivity, and vision at decreased levels of luminance since the early phases of the disease [8,9,10,11,12].

Pathogenetically, GA is increasingly recognized not only as a degenerative condition but also as an inflammatory disease. Converging evidence from genetic, histological, and preclinical studies supports chronic innate immune activation as a central mechanism in the development and progression of GA [13,14]. Recently, the first two complement inhibitors for GA treatment have been approved by the United States Food and Drug Administration (US FDA). The complement system is part of the body’s innate immune system. In the retina, innate immune functions are mediated by mononuclear phagocytes, such as microglia, monocytes and macrophages [13]. Retinal microglia, the principal resident immune cells, are key players of the innate immune system [15,16,17]. They are active sensors of the microenvironment and contribute to local complement regulation [18]. Microglia respond to injury, aging and disease by reactivity, increasing in number and modifying morphology [16,19]. Microglial reactivity is a hallmark of various retinal degenerative and inflammatory diseases, including AMD [15]. On spectral domain optical coherence tomography (SD-OCT), activated clusters of microglial cells appear as small, round hyper-reflective retinal foci with retinal nerve fiber layer (RNFL)-like reflectivity, named inflammatory HRF (I-HRF) [20,21,22]. An increased number of I-HRF was found after intraocular surgical procedure [23] and in patients suffering from neuroinflammatory diseases such as multiple sclerosis. Indeed, it has been recently demonstrated in an animal model of diabetes that focal blood–retina-barrier damage strongly associates with retinal microglia proliferation and activation, and the histologically defined pattern corresponded to the small HRF imaged by OCT [17]. Thus, I-HRF may be considered as an in vivo imaging feature of activated microglia proliferation.

Recently, we detected that I-HRF are unevenly distributed between different GA phenotypes. In patients with GA and macular new vessels (MNV) in the fellow eye (unilateral GA, U-GA), the number of I-HRF is higher than in patients with GA in both eyes (bilateral GA, B-GA) [24]. In our previous study, the mean I-HRF number, with the same features, previously obtained with the same technique in normal subjects was used as historical control. This study aimed to evaluate I-HRF distribution across inner (IR) and outer (OR) retinal layers in patients with GA compared with healthy enrolled controls.

## 2. Materials and Methods

### 2.1. Population

In this retrospective observational study, patients aged 50 years and over, affected by GA according to the Classification of Atrophy Meeting (CAM) [5], with available multimodal retinal imaging and followed at our Institution were studied. Only eyes with a total GA lesion area >1.25 mm^2^ were included. Patients were classified as bilateral GA (B-GA, both eyes analyzed) or unilateral GA (U-GA, GA eye only, fellow eye with MNV as confirmed by multimodal imaging including fluorescein and indocyanine green angiography and/or OCT-angiography). Exclusion criteria were other retinal/optic nerve diseases, MNV, macular interface disease, significant media opacity, high refractive error (±6D), recent ocular surgery (<6 months), or poor OCT quality (Q < 25). Age-matched healthy controls were also included. This study adhered to the tenets of the Declaration of Helsinki and was approved by the Ethics Committee for Clinical Practice of the Padova University Hospital. Written informed consent was obtained from all participants.

### 2.2. Imaging

All patients and controls had full ophthalmic examination including OCT, fundus autofluorescence (FAF), and near-infrared reflectance (NIR) imaging using Spectralis HRA + OCT2 platform (Heidelberg Engineering, Heidelberg, Germany). FAF and NIR images were used to quantify GA lesion area in GA eyes, with measurements obtained using the built-in software and manually verified by graders. I-HRF were defined as isolated, punctiform elements, ≤30 μm in diameter, with intermediate reflectivity similar to the retinal nerve fiber layer (RNFL) and lacking a posterior shadow cone [20].

### 2.3. Hyperreflective Retinal Foci Evaluation

**Figure 1:**
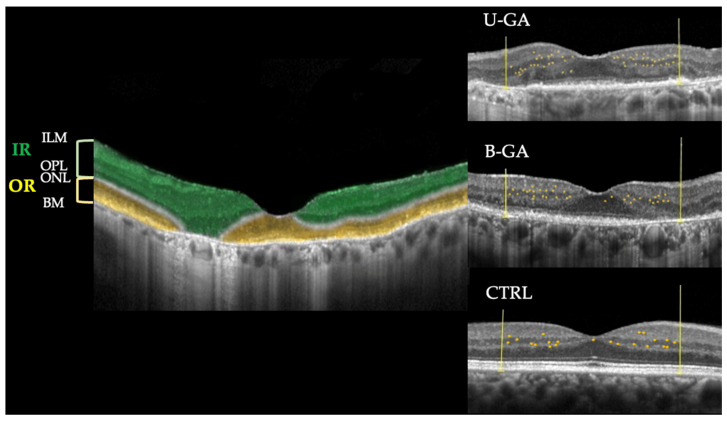
An optical coherence tomography (OCT) linear scan of a patient with geographic atrophy (GA), in which inner retinal layers (IRs) and outer retinal layer (OR) are highlighted (green for the former and yellow for the latest). On the right side of the image there are three OCT linear scans of patients with unilateral GA (U-GA), bilateral GA (B-GA) and a healthy control subject (CTRL), in which inflammatory hyperreflective retina foci (I-HRF), defined as isolated punctiform elements) of small dimensions (≤30 μm) with intermediate reflectivity (similar to that of the retinal nerve fiber layer) and without a shadow cone, are highlighted (yellow points) within the central 3 mm (within the two vertical yellow lines).

For each eye, I-HRF were manually identified and counted within the central 3 mm of the single high-resolution horizontal line scan at 180° [9 mm length, lateral resolution 12 μm/pixel, axial resolution 3.87 μm/pixel, Automatic Real-Time Tracking (ART) set at 100], spanning a region between two vertical lines located 1500 μm nasally and temporally from the foveal center. I-HRF were counted within the inner retina (IR), defined as the area between the inner limiting membrane and the lower boundary of the outer plexiform layer, and the outer retina (OR), defined as the area between the upper boundary of the outer nuclear layer and the RPE/Bruch’s membrane complex [23,25] (Figure 1).

All measurements were independently performed by two masked examiners (FP and GM), blinded to clinical data. Inter-grader agreement was evaluated using the intraclass correlation coefficient (ICC). Discrepancies were adjudicated by a senior grader (EP).

Because I-HRF counting was performed manually, reproducibility may vary across centers depending on imaging acquisition settings, grader training, and interpretation of the operational definition. In the present study, I-HRF were quantified by experienced examiners belonging to the research group that originally characterized this specific inflammatory HRF phenotype. This expertise enhances internal consistency and comparability within our cohort and reduces the risk of misclassification across the study groups; however, it also warrants caution when extrapolating this assessment to other centers, where different graders might apply slightly different subjective thresholds and obtain different counts. To mitigate this limitation, we adopted a standardized acquisition protocol and a predefined counting area, and we applied a strict, previously described operational definition (including size, reflectivity, and morphological criteria) to distinguish I-HRF from other HRFs subtypes with different imaging characteristics, as previously described by Frizziero et al. [20].

### 2.4. Statistical Analysis

Qualitative variables (e.g., sex, multifocal vs. unifocal GA, and foveal sparing) were summarized as counts and percentages. Quantitative variables (e.g., age, BCVA, GA area on NIR and FAF, and I-HRF number) were reported as mean ± standard deviation (SD). Fisher’s exact test was used for comparisons between groups, and the repeated measures ANOVA model was used to account for both eyes in the B-GA group. When an overall group effect was detected, pairwise comparisons were conducted with Tukey–Kramer adjustment for multiple comparisons. Significance was set at *p* < 0.05. Statistical analyses were performed using SAS^®^ v.9.4 (SAS Institute, Cary, NC, USA).

## 3. Results

### 3.1. Population and Geographic Atrophy Features

Sixty-eight eyes from 46 patients with GA were included: 28 patients had bilateral GA (B-GA; 49 eyes), and 19 had unilateral GA (U-GA; 19 eyes). Nineteen eyes of 19 healthy subjects served as controls. All patients and controls subjects were Caucasian, and they were homogeneous for sex distribution and age.

Mean BCVA was higher in controls than in GA eyes (85 ± 2.0 vs. 64.9 ± 21.6 ETDRS letters in U-GA, *p* = 0.002; and vs. 56.8 ± 25.7 ETDRS letters in B-GA, *p* ≤ 0.001). GA area at NIR and at FAF images did not significantly differ between the GA groups (Table 1). Mean BCVA, foveal sparing and multifocal versus unifocal GA distribution were not significantly different between GA groups (*p* > 0.05 for all). Study population demographic parameters, functional and GA clinical data are reported in Table 1.

The intergrader agreement was at least substantial for all measurements (intraclass correlation coefficient: 0.80); therefore, data from only one examiner (F.P., the one with longer experience) are reported.

### 3.2. OCT and Inflammatory Hyperreflective Retinal Foci (I-HRF)

I-HRF were significantly higher in IR than in OR across all groups (*p* < 0.001 for all). Overall, GA patients had significantly higher I-HRF in both IR and OR areas compared with controls (*p* < 0.05 for all). In U-GA, I-HRF was higher in the IR compared with B-GA (44.32 ± 8.47 vs. 30.1 ± 7.62; *p* < 0.001). The mean I-HRF in the OR, even if higher in the U-GA eyes, was not significantly different between the U-GA and B-GA groups (9.58 ± 3.04 and 8.02 ± 3.33, respectively; *p* = 0.081) (Table 2). No statistically significant correlation was detected between I-HRF count and GA area.

## 4. Discussion

GA is increasingly recognized not merely as a degenerative condition but also as a neuroinflammatory disease, in which chronic activation of the innate immune system and microglial reactivity play a central role. In this study, I-HRF were higher in IR than in the OR in all studied groups. This layering distribution pattern is consistent with I-HRF, mostly considered aggregates of activated resident microglial cells, which are predominantly localized in the IR. Physiologically, retinal microglia display a ramified homeostatic morphology, with cell bodies and processes largely distributed within the inner retinal layers and particularly along the plexiform strata, where they support synaptic function and stability [26]. In response to tissue stress, microglia can change morphology and motility, upregulate inflammatory mediators, and interact with complement-related pathways involved in AMD, potentially influencing both retinal structural reorganization and the removal of damaged cellular material [26]. Other HRF populations, such as pigmentary or exudative HRF, that differ from I-HRF in reflectivity and size are mainly localized in the OR [27,28]. This topographic distinction supports the importance of a layer-based approach when interpreting HRF on OCT, as different HRF subsets may reflect different cellular sources and microenvironmental contexts.

I-HRF were significantly higher in GA than in healthy controls. There is a well-known link between AMD and microglia activation [29]. Histopathological evidence supports an AMD-associated inflammatory response, with activated microglia alongside other cellular participants [30,31]. In this context, microglial activation may be driven by a combination of chronic oxidative stress, lipofuscin-related toxicity, complement dysregulation, and photoreceptor degeneration, all of which are relevant in AMD pathology. In GA animal models, retinal inflammation modulation can slow the progression of atrophy [32]. In human retina with signs of AMD, activated microglia associated with small HRF have been imaged with OCT as well as histology [16]. Therefore, I-HRF at OCT may serve as an in vivo imaging biomarker of retinal neuroinflammation in GA. This may be particularly relevant in the current landscape of emerging therapies targeting inflammatory and complement-related pathways, where non-invasive readouts could help monitoring strategies.

In eyes with U-GA, I-HRF were higher than in the B-GA eyes both in IR and OR, with the difference being significant in the IR. Activated microglia have been shown to shift from the inner toward the outer retinal layers and into the subretinal space, as reported in histopathological analyses [22]. This migratory behavior is consistent with the loss of polarization of microglial cells, which, in response to chronic retinal stress, tend to redistribute themselves across the retinal layers. The increased number of I-HRF may be consistent with a more pronounced pro-inflammatory state, which seems more relevant in unilateral GA eyes. Microglial plasticity is relevant to the balance between atrophic and neovascular pathways in AMD: microglia can release a broad range of inflammatory and trophic mediators, including pro-angiogenic signals, potentially promoting a pro-angiogenic environment [31]. An increase in activated microglia may reflect the higher rate of MNV developing in the GA eye of patients with MNV in the fellow eye, compared to patients with bilateral GA as previously reported by Sunness et al. [33]. Moreover, the preferential localization of I-HRF within the IR supports the idea that GA should be regarded not only as a disorder of the RPE, photoreceptor, and choriocapillaris complex but also as a neuroinflammatory disease involving the inner retina [16,26,31]. This neuroinflammatory component may contribute to the functional impairment observed in GA patients, which often exceeds what would be expected from the atrophic changes alone [11]. From a clinical standpoint, layer-specific quantification of I-HRF may contribute to a more refined phenotypic characterization of GA, potentially identifying subgroups with different neuroinflammatory activity profiles. This information could complement structural measures of atrophy enlargement and provide additional insight into disease activity. Beyond structural considerations, a layer-specific distribution of I-HRF may reflect heterogeneity in the underlying inflammatory microenvironment across GA phenotypes. Such topographic information could complement conventional atrophy-based assessment and contribute to a more nuanced interpretation of disease status. Within this conceptual framework, I-HRF emerge as a potential link between structural degeneration and immune dysregulation, reinforcing their role as a clinically meaningful OCT biomarker in GA [20,24,27]. In particular, the higher number of I-HRF observed in the IR of U-GA eyes may reflect a more intense neuroinflammatory dysregulation [24,29,31]. From a phenotypic standpoint, the differential inner retinal I-HRF burden observed between U-GA and B-GA eyes may indicate heterogeneity in inflammatory activity across GA subtypes. If confirmed in larger longitudinal cohorts, this layer-specific pattern could contribute to a more refined stratification of GA phenotypes and improve phenotypic characterization of GA subtypes. Nevertheless, the proposed links between increased I-HRF burden, heightened neuroinflammatory activity, and a pro-angiogenic environment should be regarded as interpretative hypotheses supported by prior histopathological and imaging literature [17], rather than conclusions directly demonstrated by the present dataset. The aim of our study was primarily to provide an in vivo, OCT-based topographic characterization of this specific I-HRF subtype, showing that its burden and distribution differ across GA phenotypes. These findings support the biological plausibility of the mechanisms discussed in the literature, but they do not establish causality. Prospective longitudinal studies are needed to determine whether changes in I-HRF number and layer-specific distribution over time are associated with GA progression, spatial migration of these foci, and the emergence of a pro-angiogenic microenvironment.

From a translational perspective, all these considerations may be particularly relevant in the therapeutic landscape of GA. A layer-based assessment of I-HRF distribution may provide additional insight into biological disease activity and could be particularly relevant in future interventional studies, both for baseline phenotypic characterization and for exploring potential modulation of retinal neuroinflammatory activity over time. In fact, with the recent introduction of complement-inhibiting therapies [14], there is a growing need for imaging biomarkers capable of capturing biological disease activity beyond the simple assessment of atrophy enlargement. Therefore, I-HRF may represent a valuable candidate biomarker of retinal neuroinflammation, potentially useful to monitor the biological impact of treatments targeting innate immune system pathways [13,14,20,24,26]. Changes in I-HRF number or distribution over time could provide complementary information on treatment response and inflammatory modulation, beyond evident short-term changes in atrophy growth rate [27]. Overall, the differential topographic distribution of I-HRF reinforces the concept that structural atrophy and immune-mediated retinal remodeling may coexist and variably shape the GA phenotype.

In our previous work, historical data of I-HRF obtained in healthy subjects served as controls [24]. The reference normative data were derived from a previously published cohort rather than from a prospectively enrolled, concurrently examined control group, and the control population was not strictly age-matched to the GA cohort. That methodological aspect may have introduced systematic bias and limited statistical power, thereby reducing the sensitivity to detect differences between B-GA eyes and healthy controls [24]. In the present study, the inclusion of a contemporaneously enrolled, age-matched control cohort examined using the same acquisition protocol and grading methodology represents a meaningful methodological refinement. This approach minimizes potential confounding related to demographic variability, imaging settings, and grading procedures, thereby strengthening the internal validity of the comparison. The confirmation of a significantly higher I-HRF burden in GA eyes under identical imaging conditions enhances the robustness of our findings and supports the reproducibility of the observed phenotype-specific differences.

The main limitation of this study is the manual count of I-HRF on OCT scans which is time-consuming and requires expert and well-trained operators. Additionally, manual identification may be challenged by borderline hyperreflective elements and by the need to consistently distinguish I-HRF from other small hyperreflective OCT features, underscoring the value of standardized grading criteria. In addition, the use of a single HR linear scan may not fully capture the three-dimensional spatial distribution of I-HRF across the macula and may therefore underestimate the heterogeneity of neuroinflammatory activity. Automatic methods for I-HRF quantifications, recently available, will allow us to easily obtain normative datasets and automated, standardized quantification pipelines to enhance the robustness and reproducibility of I-HRF assessment [34]. Automated approaches could also enable future longitudinal studies exploring clinical correlations between I-HRF and GA rate progression across GA different phenotypes. Another limitation relates to the relatively small size of the U-GA subgroup, which may have reduced statistical power, particularly for OR comparisons. Notably, despite the limited sample size, the difference in IR I-HRF burden between U-GA and B-GA eyes remained statistically significant, underscoring the robustness of this layer-specific finding. In contrast, although a higher OR I-HRF burden was observed in U-GA eyes, the difference did not reach statistical significance and may reflect insufficient power rather than a true absence of effect.

## 5. Conclusions

In conclusion, I-HRF are higher in eyes with GA than in age-matched healthy controls, supporting the role of neuroinflammation in GA, mainly in specific GA phenotypes. I-HRF might serve as valuable biomarkers for diagnosing, monitoring, and predicting disease progression. Further prospective longitudinal studies and larger populations are essential to address the clinical and pathogenetic significance of I-HRF in GA, mainly considering the advent of new therapeutic strategies targeting neuroretinal inflammation.

## Figures and Tables

**Table 1:** Study population demographic parameters, Best Corrected Visual Acuity (BCVA) and geographic atrophy clinical features.

Parameter	B-GA Group (n = 28 Patients, 49 Eyes)	U-GA Group (n = 19 Patients, 19 Eyes)	Controls (n = 19 Healthy Subjects, 19 Eyes)	*p*-Value
Sex males:females number (%)	5:23 (18:82)	4:15 (21:79)	10:9 (53:47)	0.156
Age (years) mean age ± SD (range)	82.6 ± 7.6 (70–94)	84.3 ± 6.3 (74–95)	81.1 ± 5.1 (65–89)	* 0.095 ^†^ 0.446 ** 0.4
BCVA (ETDRS letters) mean ± SD (range)	56.8 ± 25.7 (3–85)	64.9 ± 21.6 (3–85)	85 ± 2.0 (83–87)	** 0.197 * 0.002 **^†^ <0.001**
GA area in NIR (mm^2^) mean age ± SD (range)	10.7 ± 7.34 (0.83–34.5)	9.74 ± 10.4 (0.57–30.8)	n.a.	0.673
GA area in FAF (mm^2^) mean age ± SD (range)	9.88 ± 7.08 (0.59–32.9)	9.44 ± 10.41 (0.39–30.0)	n.a.	0.533
Multifocal vs. Unifocal number (%)	21:28 (43:57)	11:8 (58:42)	n.a.	0.292
Foveal sparing (yes:no) number (%)	20:29 (40:60)	13:6 (63:37)	n.a.	0.285

GA: geographic atrophy; B-GA group: patients with bilateral geographic atrophy; U-GA group: patients with geographic atrophy in one eye and macular new vessels in the fellow eye; NIR: near-infrared reflectance; FAF: fundus autofluorescence; n: number; SD: standard deviation. Significant *p*-values are in bold. * U-GA vs. controls; ^†^ B-GA vs. controls; ** U-GA vs. B-GA; n.a.: not applicable.

**Table 2:** Inflammatory HRF distribution in inner and outer retina.

HRF	B-GA Group (n = 49 Eyes)	U-GA Group (n = 19 Eyes)	Controls (n = 19 Eyes)	*p*-Value
Inner retina (IR) mean age ± SD	30.10 ± 7.62	44.32 ± 8.47	25.11 ± 4.98	<0.001 * 0.010 **
Outer retina (OR) mean age ± SD	8.02 ± 3.33	9.58 ± 3.04	3.63 ± 2.03	0.081 ^†^ <0.001 *^†^
*p*-value (IR vs. OR)	**<0.001**	**<0.001**	**<0.001**	

IR: Inner retina; OR: outer retina; GA: geographic atrophy; B-GA group: patients with bilateral geographic atrophy (GA); U-GA group: patients with GA in one eye and macular new vessels in the fellow eye; n: number; SD: standard deviation; Significant *p*-values are in bold. * U-GA vs. B-GA and U-GA vs. controls; ** B-GA vs. controls; ^†^ U-GA vs. B-GA; *^†^ B-GA vs. controls and U-GA vs. controls.

## Data Availability

The original contributions presented in this study are included in the article. Further inquiries can be directed to the corresponding author.

## Abbreviations

The following abbreviations are used in this manuscript:

AMD	Age-related Macular Degeneration
GA	Geographic Atrophy
I-HRF	Inflammatory Hyperreflective Retinal Foci
OCT	Optical Coherence Tomography
IR	Inner Retina
OR	Outer Retina
B-GA	Bilateral Geographic Atrophy
U-GA	Unilateral Geographic Atrophy
MNV	Macular Neovascularization
FAF	Fundus Autofluorescence
NIR	Near-Infrared Reflectance
ART	Automatic Real-Time Tracking
RNFL	Retinal Nerve Fiber Layer
CAM	Classification of Atrophy Meeting
BCVA	Best Corrected Visual Acuity
